# Supplementary material for: Comprehensive Exonic Sequencing of Known Ataxia Genes in Episodic Ataxia
Source: Biomedicines. 2020 May 25;8(5):134. doi: 10.3390/biomedicines8050134 (PMC7277596; doi:10.3390/biomedicines8050134)
Supplement: Supplementary file 1 [file biomedicines-08-00134-s001.zip › biomedicines-802193-supplementary-fianl/Supplementary Table S1.pdf]

Supplementary Table S1: 170 ataxia-gene panel used in Tier 1 analysis.

Autosomal Dominant genes		Autosomal recessive genes		X-linked genes	Disorders with Spasticity and Cerebellar Ataxia	Speculative	Joubert	Other disorders associated with Ataxia	Ataxia in mouse models	tRNA synthetase genes
**Episodic Ataxia genes**	**SC Ataxia genes**	**AR genes reported in >5 families**	**AR genes reported in <5 families**							
KCNA1 (EA1)	ATXN1	ANO10	ABHD12	ABCB7	VAMP1	AIFM1	AHI1	PRPS1	AARS	AARS2
CACNA1A	ATXN2	APTX	ACO2	ATP2B3	KIF1C	EN1	ARL13B	GAN	CHD6	CARS
CACNB4	ATXN3	ATM	COQ8A	CASK	MARS2	EN2	CC2D2A	SLC6A19	A2BP1/ RBFOX1	CARS2
SLC1A3	PLEKHG4	C10orf2	ATCAY	FMR1	MTPAP	KLHL1	CEP290	TINF2	ATP2B2	DARS
SCN2A	SPTBN2	CYP27A1	ATG5	OPHN1	AFG3L2	RAB3A	INPP5E	PSAP	AGTPBP1	DARS2
ATP1A3	ATXN7	FXN	ATP8A2	SLC9A6	SPG7	SYNE2	NPHP1	SCN1A	DNAJC19	EARS2
UBR4	ATXN8	PHYH, PEX7	CAPN1			EEF2	OFD1		DST	EPRS
	ATXN8OS	PNPLA6	CLCN2				RPGRIP1 L		GRID2	FARS2
	ATXN10	RFC1	CLN5				TMEM21 6		HERC1	FARSA
	TTBK2	SACS	CWF19L1				TMEM67		KCNJ6	FARSB
	PPP2R2B	SETX	FLVCR1						MYO5A	GARS
	KCNC3	SIL1	GDAP2						RELN	HARS
	PRKCG	SLC52A2	GOSR2						RORA	HARS2
	ITPR1	SNX14	GRID2						SLC12A6	IARS
	TBP	SYNE1	GRM1						SNAP25	IARS2
	FGF14	TTPA	KCNJ10						TRPC3	KARS
	AFG3L2	WFS1	WWOX						USP14	LARS
	BEAN1	MRE11A							AFF1	LARS2
	KCND3	TDP1								MARS

	TMEM240	CABC1/								NARS
	PDYN	MARS2								NARS2
	SCA25	KIAA0226								PARS2
	EEF2	DARS2								QARS
	ELOVL4	AAAS								RARS
	TGM6	VLDLR								RARS2
	NOP56									SARS
	ELOVL5									SARS2
	CCDC88C									TARS
	TRPC3									TARS2
	CACNA1G									VARS
	MME									VARS2
	GRM1									WARS
	FAT2									WARS2
	PLD3									YARS
	PUM1									YARS2
	STUB1									
	ATN1									
	DNMT1									
	TUBB4A									
	C9orf72									
	FGF12									
