# Supplementary material for: Comprehensive Exonic Sequencing of Known Ataxia Genes in Episodic Ataxia
Source: Biomedicines. 2020 May 25;8(5):134. doi: 10.3390/biomedicines8050134 (PMC7277596; doi:10.3390/biomedicines8050134)
Supplement: Supplementary file 1 [file biomedicines-08-00134-s001.zip › biomedicines-802193-supplementary-fianl/Supplementary Table S2.pdf]

Supplementary Table S2: The 353 ion channel genes used in Tier 2 analysis.

SCNN1G	CACNA1C-AS2	ATP6V0E1	ATP6V1D	ATP5LP2	ATP5G1P7	KCNH7	KCND3-IT1	KCNJ4	CLCC1
SCN10A	CACNA1C-AS1	ATP6V1G2	ATP6V1C1	ATP5LP3	ATP5G1P6	KCNK12	KCNK15-AS1	KCNJ5	CLCA4
SCNM1	CACNA1C-AS4	ATP11C	ATP6V1C2	ATP7A	ATP6AP1L	KCNH8	KCNK5	KCNJ6	
SCNN1D	CACNA1C-AS3	ATP1B4	ATP6V1F	ATP5HP1	ATP6V1G3	KCNK13	KCNK6	KCNJ10	
SCN11A	CACNA2D1	ATP1B3P1	ATP6V0A1	ATP6V0E2-AS1	KCNH1-IT1	KCNK15	KCNK7	KCNIP1	
SCN8A	CACNA2D3	ATP11B	ATP5F1P3	ATPAF2	KCNE1	KCNK16	KCNK9	KCNJ11	
SCNN1B	CACNA2D2	ATP1B3	ATP5F1P4	ATP6V0B	KCNE2	KCNK17	KCNC1	KCNJ8	
SCN9A	CACNA2D4	ATP5J2P3	ATP5F1P1	ATP6AP1	ATP8A2P2	KCNK18	KCNC2	KCNJ9	
SCNN1A	CACNB1	ATP11A	ATP8B4	ATP11A-AS1	ATP8A2P3	KCNQ5-AS1	KCNC3	KCNIP2	
SCN7A	CACNB2	ATP1B2	ATP5F1P2	ATPIF1	ATP8A2P1	KCNQ1	KCNC4	KCNJ12	
SCN4A	CACNB3	ATP1B1	ATP8B3	ATP6AP2	ATP13A1	KCNQ2	KCNAB1-AS2	KCNB1	
SCN5A	CACNB4	ATP6V0E1P2	ATP8B2	ATP1B1P1	ATP2B4	KCNQ3	KCNAB1-AS1	KCNIP3	
SCN2A	CACNA1C-IT3	ATP6V0E1P1	ATP8B1	ATP5F1	ATP2B3	KCNQ4	KCNT1	KCNJ13	
SCN3B	CACNA1C-IT2	ATP6V0E1P4	ATP1A1-AS1	ATP6C	ATP2B2	KCNQ5	KCNT2	KCNB2	
SCN3A	CACNA1C-IT1	ATP5J2P6	ATP5F1P7	ATP2A1-AS1	ATP2B1	KCNQ1DN	KCND1	KCNIP4	
SCN4B	ATP5G2P4	ATP6V0E1P3	ATP5F1P5	ATP6V1B2	ATP6V1B1-AS1	KCNA10	KCND2	KCNJ14	
SCN1B	ATP5G2P3	ATP5J2	ATP5F1P6	ATP6V1E1P1	ATP13A4	KCNA1	KCND3	KCNJ15	
SCN1A	ATP5G2P1	ATP2B2-IT2	ATP4B	ATP6V0C	ATP13A5	KCNE3	KCNAB1	KCNJ16	
SCN2B	ATP8A2	ATP2B2-IT1	ATP4A	ATPAF1	ATP13A2	KCNE4	KCNAB2	KCNRG	
CACNA2D3-AS1	ATP8A1	ATP6V0D1	ATP5J2-PTCD1	ATP6V1B1	ATP1B3-AS1	KCNE5	KCNAB3	KCNJ18	
CACNA1B	ATP5BP1	ATP6V0D2	ATP6V1G1P6	ATP5G1P5	ATP6V1G2-DDX39B	KCNMB2-AS1	KCNJ2-AS1	KCNS1	
CACNA1A	ATP5A1P5	ATP6V1A	ATP6V1G1P7	ATP5G1P4	ATP13A3	KCNQ1-AS1	KCNIP2-AS1	KCNS2	
CACNA1D	ATP5C1	ATP5S	ATP6V1G1P2	ATP5G1P3	ATP6V0CP1	KCNE1B	KCNU1	KCNIP4-IT1	
CACNA1C	ATP5A1P6	ATP5C1P1	ATP6V1G1P3	ATP5EP1	ATP6V0CP3	KCNC4-AS1	KCNMA1	KCNS3	
CACNA1F	ATP5A1P7	ATP1A1OS	ATP6V1G1P4	ATP5EP2	ATP6V0CP2	KCNV1	CLCP2	KCNK1	
CACNA1E	ATP12A	ATP5J	ATP2A3	ATP5G1P1	KCNG1	KCNV2	CLCN7	KCNK2	
CACNA1H	ATP5A1P1	ATP13A5-AS1	ATP2A2	ATP13A4-AS1	KCNG2	KCNMB1	CLCN6	KCNK3	
CACNA1G	ATP5A1P2	ATP5I	ATP2A1	ATP23	KCNG3	KCNQ1OT1	KCNMB3P1	KCNK4	

CACNG6	ATP8B5P	ATP5H	ATP5G3	ATP10D	KCNG4	KCNMB2	KCNA2	CLCA3P	
CACNG7	ATP5A1P3	ATP5O	ATP5G2	ATP5A1	KCNH1	KCNMB3	KCNA3	CLCNKB	
CACNA1I	ATP5L2	ATP5J2LP	ATP5G1	ATP1A4	KCNH2	KCND3-AS1	KCNQ5-IT1	CLCNKA	
CACNG8	ATP5A1P8	ATP5L	ATP7B	ATP10B	KCNMA1-AS3	KCNMB4	KCNA4	CLCN3	
CACNA1G-AS1	ATP5A1P9	ATP5B	ATP5LP4	ATP1A3	KCNH3	KCNN1	KCNA5	CLCN2	
CACNA1S	ATP11AUN	ATP5A1P10	ATP6V0E2	ATP1A2	KCNMA1-AS2	KCNN2	KCNA6	CLCN1	
CACNG1	ATP6V1E1	ATP5E	ATP9B	ATP10A	KCNH4	KCNE1L	KCNA7	CLCN5	
CACNG2	ATP6V1E2	ATP5D	ATP9A	ATP1A1	KCNMA1-AS1	KCNN3	KCNJ6-IT1	CLCN4	
CACNG3	ATP5SL	ATP6V0A2	ATP5LP5	ATP2C2	KCNH5	KCNN4	KCNJ1	CLCF1	
CACNG4	ATP5JP1	ATP6V1H	ATP5LP6	ATP2C1	KCNK10	KCNF1	KCNJ2	CLCA2	
CACNG5	ATP6V1G1	ATP6V0A4	ATP5LP7	ATP5G1P8	KCNH6	KCNK4-TEX40	KCNJ3	CLCA1	
