# Supplementary material for: Comprehensive Exonic Sequencing of Known Ataxia Genes in Episodic Ataxia
Source: Biomedicines. 2020 May 25;8(5):134. doi: 10.3390/biomedicines8050134 (PMC7277596; doi:10.3390/biomedicines8050134)
Supplement: Supplementary file 1 [file biomedicines-08-00134-s001.zip › biomedicines-802193-supplementary-fianl/Supplementary Table S3.pdf]

Supplementary Table S3: Variants identified in 170 ataxia-gene panel.

ID#	Locus	Ref	Genes	Amino Acid Change	Coding	gnomAD frequency	Transcript	ACMG rules	verdict
1	chr1:19500021	T	UBR4	p.Asn1026Ile	c.3077T>A	-	NM_020765.2	PM2,BP1	Uncertain Significance
1	chr3:63965786	G	ATXN7	p.Arg232Lys	c.695G>A	0.000317	NM_001177387.1	BS1, BS2, BP1, BP4	Benign
1	chr19:3977995	T	EEF2	p.Gln630Arg	c.1889A>G	-	NM_001961.3	PM2, BP1, BP4	Likely Benign
**2**	**chr2:166231195**	**G**	**SCN2A**	**p.Val1325Phe**	**c.3973G>T**	**-**	**NM_001040143.1**	**PM1, PM2, PP2, PP3**	**Likely Pathogenic**
2	chr5:150887120	C	FAT2	p.Glu4038Lys	c.12112G>A	0.0009	NM_001447.2	BS1, BS2, BP1, BP4	Benign
4	chr4:88048187	G	AFF1	p.Gly941Arg	c.2821G>A	0.0000119	NM_001166693.2	PM2, BP1, BP4	Likely Benign
4	chr14:64469660	C	SYNE2	p.Leu1337Val	c.4009C>G	0.0000161	NM_182914.2	PM2, BP1, BP4	Likely Benign
4	chr14:64691206	G	SYNE2	p.Asp6804Asn	c.20410G>A	0.00279	NM_182914.2	BS1, BS2, BP1, BP4, BP6	Benign
4	chr16:70293064	T	AARS	p.Asn604Ser	c.1811A>G	0.000166	NM_001605.2	PP3, BS1, BP1	Likely Benign
6	chr6:152630996	C	SYNE1	p.Ala5726Thr	c.17176G>A	0.0000159	NM_182961.3	PM2,BP4	Uncertain Significance
6	chr9:88248021	T	AGTPBP1	p.His484Arg	c.1451A>G	0.00012	NM_015239.2	BS1, BP4	Likely Benign
6	chr17:4923867	G	KIF1C	p.Pro614fs	c.1832_1833insT	-	NM_006612.5	PM2, PP3	Uncertain Significance
**6**	**chr19:42474691**	**C**	**ATP1A3**	**p.Arg756His**	**c.2267G>A**	**-**	**NM_152296.4**	**PM1, PM2, PM5, PP2, PP3, PP5**	**Pathogenic**
7	chr19:39421947	G	SARS2, MRPS12	p.Gly5Ser	c.13G>A	0.000518	NM_021107.1	BS1, BP4	Likely Benign
10	chr1:31532260	C	PUM1	p.Ala52Ser	c.154G>T	-	NM_001020658.1	PM2, BP4	Uncertain Significance
10	chr20:2411674	G	TGM6	p.?	c.1967+1G>A	0.0000199	NM_198994	PVS1,PP3, BS2	Uncertain Significance
11	chr7:30639618	C	GARS	p.Thr127Asn	c.380C>A	0.00000801	NM_002047.3	PM2, PP3, BP1	Uncertain Significance
14	chr14:64574288	G	SYNE2	p.Gly4191Asp	c.12572G>A	0.000863	NM_182914.2	BS1, BS2, BP1, BP4, BP6	Benign
16	chr16:7568263	C	RBFOX1	p.Pro68Ser	c.202C>T	0.000633	NM_145891.2	PP3, BS1, BP1,BP6	Likely Benign
16	chrX:13765031	C	OFD1	p.Arg263Trp	c.787C>T	0.0000109	NM_003611.2	PM2,PP3	Uncertain Significance
16	chrX:135126765	G	SLC9A6	p.Arg663Gln	c.1988G>A	0.0000273	NM_001042537.1	BS1, BS2, BP1, BP4	Benign
19	chr6:30893459	C	VARS2	p.Pro1005Leu	c.3014C>T	0.0000416	NM_001167734.1	BP1	Uncertain Significance
19	chr6:56323828	T	DST	p.Lys5668Arg	c.17003A>G	0.000329	NM_001144769.2	BS1, BP1, BP4	Likely Benign
**19**	**chr6:137143923**	**C**	**PEX7**	**p.Tyr40Ter**	**c.120C>G**	**0.0000907**	**NM_000288.3**	**PVS1,PM2, PP3, PP5**	**Pathogenic**
19	chr7:103206742	C	RELN	p.Arg1622Gln	c.4865G>A	0.000012	NM_173054.2	PM2, PP3, BP1	Uncertain Significance
20	chr6:86282026	T	SNX14	p.Lys110Glu	c.328A>G	0.000361	NM_153816.5	BS1, BP1, BP4	Likely Benign

20	chr6:152755013	C	SYNE1	p.Val1460Ile	c.4378G>A	0.000156	NM_182961.3	BS1,BS2, BP4	Benign
21	chr13:23905843	T	SACS	p.Arg4058Gly	c.12172A>G	0.0000177	NM_014363.5	PM2, BP1, BP4	Likely Benign
21	chr14:91780445	G	CCDC88C	p.Ser572Leu	c.1715C>T	0.000351	NM_001080414.3	PP3, BS1, BP1	Likely Benign
21	chr15:63946518	G	HERC1	p.Leu3364Val	c.10090C>G	0.0000164	NM_003922.3	PM2, BP1, BP4	Likely Benign
21	chr16:23540823	C	EARS2	p.Gly451Val	c.1352G>T	0.0000403	NM_001083614.1	PM2, PP2, BP4	Uncertain Significance
21	chr16:89590603	G	SPG7	p.Ser189Asn	c.566G>A	0.000113	NM_003119.3	PP2, BP4	Uncertain Significance
23	chr6:152711445	T	SYNE1	p.Asp2716Gly	c.8147A>G	0.0000199	NM_182961.3	BS2, BP4	Likely Benign
**23**	**chr12:5021044**	**G**	**KCNA1**	**p.Arg167Met**	**c.500G>T**	**-**	**NM_000217.2**	**PM1, PM2, PP2, PP3**	**Likely Pathogenic**
25	chr1:220267800	C	IARS2	p.Pro81Leu	c.242C>T	0.0000131	NM_018060.3	PM2, BP1, BP4	Likely Benign
25	chr2:219677434	A	CYP27A1	p.Lys269Arg	c.806A>G	-	NM_000784.3	PM2, PP2, BP4	Uncertain Significance
25	chr20:1961350	C	PDYN	p.Glu128Asp	c.384G>T	-	NM_001190898	PM2, BP1, BP4	Benign
31	chr13:23905549	G	SACS	p.Pro4156Ser	c.12466C>T	0.00000398	NM_014363.5	PM2, PP3, BP1	Uncertain Significance

American College of Medical Genetics and Genomics; VOUS: variants of unknown significance; NA: not applicable. Variants reported in the manuscript are in **bold**
