# Supplementary figures and images for: Comprehensive Exonic Sequencing of Known Ataxia Genes in Episodic Ataxia
Source: Biomedicines. 2020 May 25;8(5):134. doi: 10.3390/biomedicines8050134 (PMC7277596; doi:10.3390/biomedicines8050134)

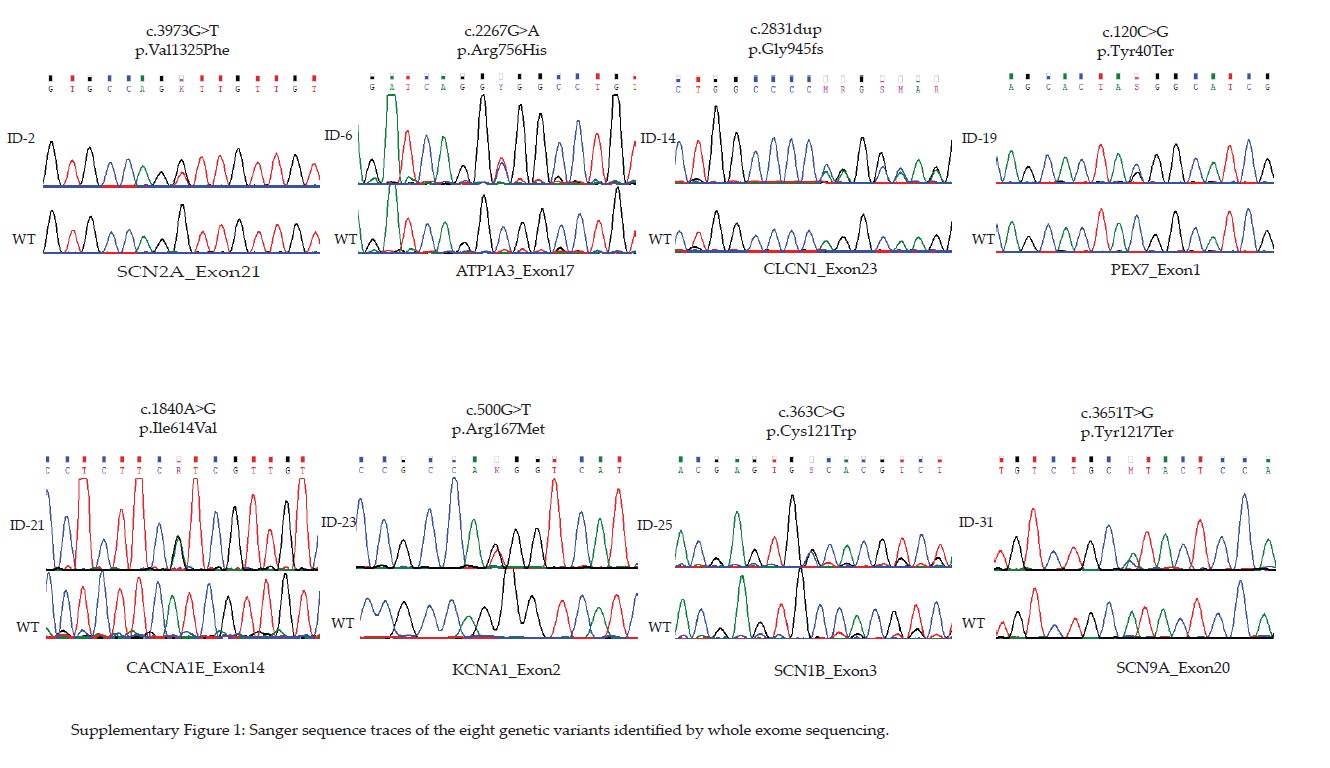

Supplement: Supplementary file 1 [file biomedicines-08-00134-s001.zip › biomedicines-802193-supplementary-fianl/Figure S1.jpg]

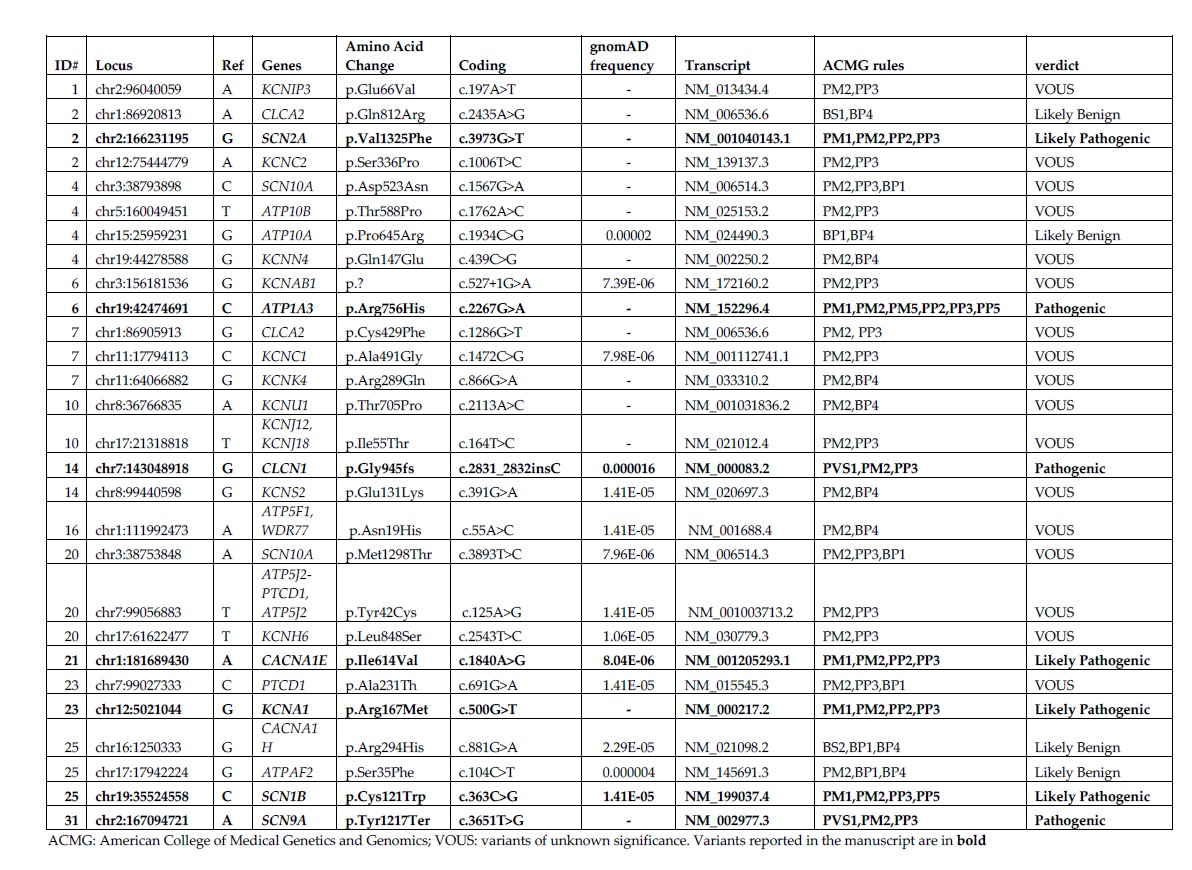

Supplement: Supplementary file 1 [file biomedicines-08-00134-s001.zip › biomedicines-802193-supplementary-fianl/Table S4.jpg]
